# Supplementary material for: Meta-Analysis and Gene Set Enrichment Relative to ER Status Reveal Elevated Activity of MYC and E2F in the “Basal” Breast Cancer Subgroup
Source: PLoS One. 2009 Mar 9;4(3):e4710. doi: 10.1371/journal.pone.0004710 (PMC2650420; doi:10.1371/journal.pone.0004710)
Supplement: Dataset S1 — GSEA results from first screen. The file contains a zipped archive of the HTML results of the second GSEA screen. Reports for enriched or depleted datasets may be accessed from the “index.html” link within the my_analysis.GseaPreranked.1219975950625.rpt.zip ZIP archive. Enrichment plots are available for each of the 50 most enriched and depleted gene sets. (13.32 MB ZIP) [file pone.0004710.s001.zip › my_analysis.GseaPreranked.1219975950625.rpt.zip!//ADIP_DIFF_CLUSTER5.html]

Details for gene set ADIP\_DIFF\_CLUSTER5[GSEA]

|  || Dataset | zSco.ERneg.to.ERpos\_collapsed |
| Phenotype | NoPhenotypeAvailable |
| Upregulated in class | na\_pos |
| GeneSet | ADIP\_DIFF\_CLUSTER5 |
| Enrichment Score (ES) | 0.72539485 |
| Normalized Enrichment Score (NES) | 2.4582129 |
| Nominal p-value | 0.0 |
| FDR q-value | 0.0 |
| FWER p-Value | 0.0 |
Table: GSEA Results Summary

  

Fig 1: Enrichment plot: ADIP\_DIFF\_CLUSTER5      
 Profile of the Running ES Score & Positions of GeneSet Members on the Rank Ordered List

  

| PROBE | GENE SYMBOL | GENE\_TITLE | RANK IN GENE LIST | RANK METRIC SCORE | RUNNING ES | CORE ENRICHMENT || 1 | CDC20 |  |  | 9 | 7.151 | 0.0614 | Yes |
| 2 | BUB1 |  |  | 41 | 6.445 | 0.1150 | Yes |
| 3 | CCNA2 |  |  | 52 | 6.297 | 0.1689 | Yes |
| 4 | AURKB |  |  | 76 | 6.038 | 0.2195 | Yes |
| 5 | SLPI |  |  | 140 | 5.641 | 0.2637 | Yes |
| 6 | MELK |  |  | 181 | 5.424 | 0.3078 | Yes |
| 7 | PTTG1 |  |  | 225 | 5.206 | 0.3498 | Yes |
| 8 | STMN1 |  |  | 310 | 4.907 | 0.3860 | Yes |
| 9 | KIF20A |  |  | 318 | 4.874 | 0.4278 | Yes |
| 10 | PRC1 |  |  | 543 | 4.253 | 0.4478 | Yes |
| 11 | RANGAP1 |  |  | 712 | 3.909 | 0.4691 | Yes |
| 12 | NUSAP1 |  |  | 836 | 3.732 | 0.4923 | Yes |
| 13 | RANBP5 |  |  | 838 | 3.729 | 0.5245 | Yes |
| 14 | H2AFX |  |  | 930 | 3.591 | 0.5489 | Yes |
| 15 | UBE2C |  |  | 994 | 3.489 | 0.5744 | Yes |
| 16 | CAD |  |  | 1051 | 3.408 | 0.5998 | Yes |
| 17 | CDC2 |  |  | 1259 | 3.182 | 0.6118 | Yes |
| 18 | S100A8 |  |  | 1444 | 3.003 | 0.6240 | Yes |
| 19 | C9ORF16 |  |  | 1524 | 2.925 | 0.6434 | Yes |
| 20 | HMMR |  |  | 1608 | 2.843 | 0.6619 | Yes |
| 21 | CCNB1 |  |  | 1626 | 2.823 | 0.6851 | Yes |
| 22 | ELAVL1 |  |  | 1751 | 2.727 | 0.6994 | Yes |
| 23 | CDC25C |  |  | 1794 | 2.700 | 0.7197 | Yes |
| 24 | EXOSC5 |  |  | 2010 | 2.523 | 0.7254 | Yes |
| 25 | LCN2 |  |  | 2658 | 2.113 | 0.6950 | No |
| 26 | TK1 |  |  | 2730 | 2.076 | 0.7077 | No |
| 27 | RACGAP1 |  |  | 4097 | 1.475 | 0.6177 | No |
| 28 | RRM1 |  |  | 4872 | 1.197 | 0.5698 | No |
| 29 | AQP1 |  |  | 7776 | 0.199 | 0.3531 | No |
| 30 | KIF22 |  |  | 8725 | -0.180 | 0.2833 | No |
| 31 | SFRS1 |  |  | 8891 | -0.255 | 0.2731 | No |
| 32 | GALK1 |  |  | 9378 | -0.495 | 0.2408 | No |
| 33 | AGT |  |  | 10424 | -1.102 | 0.1717 | No |
| 34 | XTP3TPA |  |  | 10592 | -1.236 | 0.1698 | No |
| 35 | ISYNA1 |  |  | 12877 | -4.080 | 0.0333 | No |
Table: GSEA details [plain text format]

  

Fig 2: ADIP\_DIFF\_CLUSTER5: Random ES distribution      
 Gene set null distribution of ES for **ADIP\_DIFF\_CLUSTER5**
